# Low Levels of Natural Anti-α-*N*-Acetylgalactosamine (Tn) Antibodies Are Associated With COVID-19

**DOI:** 10.3389/fmicb.2021.641460

**Published:** 2021-02-11

**Authors:** Adrien Breiman, Nathalie Ruvoën-Clouet, Marie Deleers, Tiffany Beauvais, Nicolas Jouand, Jézabel Rocher, Nicolai Bovin, Nathalie Labarrière, Hanane El Kenz, Jacques Le Pendu

**Affiliations:** ^1^Université de Nantes, INSERM, CRCINA, Nantes, France; ^2^CHU de Nantes, Nantes, France; ^3^Oniris, Ecole Nationale Vétérinaire, Agroalimentaire et de l’Alimentation, Nantes, France; ^4^Department of Transfusion, CHU Brugmann, Université Libre de Bruxelles (ULB), Brussels, Belgium; ^5^Laboratory of Immunology, LHUB-ULB, Brussels, Belgium; ^6^Platform Cytocell, SFR François Bonamy, Nantes, France; ^7^Shemyakin-Ovchinnikov Institute of Bioorganic Chemistry, Russian Academy of Sciences, Moscow, Russia

**Keywords:** COVID-19, *O*-glycans, natural antibodies, Tn antigen, histo-blood group antigens

## Abstract

Human serum contains large amounts of anti-carbohydrate antibodies, some of which may recognize epitopes on viral glycans. Here, we tested the hypothesis that such antibodies may confer protection against COVID-19 so that patients would be preferentially found among people with low amounts of specific anti-carbohydrate antibodies since individual repertoires vary considerably. After selecting glycan epitopes commonly represented in the human anti-carbohydrate antibody repertoire that may also be expressed on viral glycans, plasma levels of the corresponding antibodies were determined by ELISA in 88 SARS-CoV-2 infected individuals, including 13 asymptomatic, and in 82 non-infected controls. We observed that anti-Tn antibodies levels were significantly lower in patients as compared to non-infected individuals. This was not observed for any of the other tested carbohydrate epitopes, including anti-αGal antibodies used as a negative control since the epitope cannot be synthesized by humans. Owing to structural homologies with blood groups A and B antigens, we also observed that anti-Tn and anti-αGal antibodies levels were lower in blood group A and B, respectively. Analyses of correlations between anti-Tn and the other anti-carbohydrates tested revealed divergent patterns of correlations between patients and controls, suggesting qualitative differences in addition to the quantitative difference. Furthermore, anti-Tn levels correlated with anti-S protein levels in the patients’ group, suggesting that anti-Tn might contribute to the development of the specific antiviral response. Overall, this first analysis allows to hypothesize that natural anti-Tn antibodies might be protective against COVID-19.

## Introduction

Viral envelope proteins, including those of the severe acute respiratory syndrome coronavirus 2 SARS-CoV-2 are extensively glycosylated (Watanabe et al., 2020). Since these glycans are synthesized by the host cell enzymatic machinery, they are part of the self and have little immunogenic potential. Alongside other functions, the glycan shield masks the protein surface from potential peptide specific antibodies (Bagdonaite and Wandall, 2018). Glycosylation is therefore exploited by enveloped viruses as a protection mechanism (Watanabe et al., 2019). Yet, it might also constitute a Trojan horse. Indeed, several carbohydrate antigenic epitopes may be present on viral envelope glycoproteins. The αGal antigen is the most extensively studied example of a carbohydrate epitope that can lead to the elimination of viruses through natural antibodies (Galili, 2019). This carbohydrate antigen is expressed by many cell types in most mammalian species, but is lacking in humans, apes and old-world monkeys due to pseudogenization of the *GGTA1* gene that encodes the galactosyltransferase required for its synthesis. As a result, species unable to express the αGal antigen produce natural anti-αGal antibodies in response to bacteria of the microbiota that carry mimicking carbohydrate structures. It has been established that several types of enveloped viruses, including influenza virus, murine C retrovirus, porcine endogenous retrovirus, lymphocytic choriomeningitis virus, Newcastle disease virus, Sindbis virus, vesicular stomatitis virus, measles virus, and paramyxovirus present the αGal antigen when produced in cells that synthesize it (Galili, 2020). Anti-αGal antibodies can directly neutralize these viruses or opsonize them leading to complement-mediated destruction or to amplification of the immune response by targeting antigen presenting cells. It is thus believed that these xenogenic natural antibodies contribute to protect our species from zoonotic transmission of enveloped viruses (Galili, 2019). Likewise, enveloped viruses can be decorated with allogeneic carbohydrate epitopes of the ABO blood group type. Thus, measles viruses produced by cells expressing either the A or B blood group antigens was neutralized by the natural cognate antibodies in a complement-dependent manner (Preece et al., 2002). Moreover, anti-A antibodies could block the interaction between SARS-CoV S protein and its cellular receptor, the angiotensin-converting enzyme ACE2, when the viral protein was produced by cells expressing the A blood group antigen (Guillon et al., 2008). This was consistent with the expression of blood group antigens by respiratory tract epithelial cells where the virus replicates and the lesser risk of infection of blood group O individuals by SARS-CoV observed in a Hong Kong hospital outbreak (Cheng et al., 2005). Indeed, group O individuals possess anti-A and anti-B antibodies that could have protected them from viral particles emitted by either blood group A or B patients. Interestingly, a large number of observations indicate that blood group O individuals have a lower risk of COVID-19, whereas blood group A individuals appear to be at a higher risk (Cheng et al., 2005; Abdollahi et al., 2020; Ahmed et al., 2020; Aljanobi et al., 2020; Barnkob et al., 2020; Chegni et al., 2020; Delanghe et al., 2020; Dzik et al., 2020; Ellinghaus et al., 2020; Fan et al., 2020; Franchini et al., 2020; Gallian et al., 2020; Göker et al., 2020; Hoiland et al., 2020; Latz et al., 2020; Leaf et al., 2020; Li et al., 2020; Muniz-Diaz et al., 2020; Niles et al., 2020; Padhi et al., 2020; Ray et al., 2020; Roberts et al., 2020; Shelton et al., 2020; Sohlpour et al., 2020; Valenti et al., 2020; Wu et al., 2020; Zeng et al., 2020; Zhang et al., 2020; Zhao J. et al., 2020; Zietz et al., 2020). Only a few studies failed to find any association between ABO types and COVID-19, likely depending on study design (Boudin et al., 2020; Focosi et al., 2020; Pairo-Castineira et al., 2020). Coherent with the notion that natural anti-carbohydrate could have a protective effect, we recently observed that COVID-19 patients present lower levels of anti-A and/or anti-B blood group antibodies than controls (Deleers et al., 2020).

In addition to anti-xenogenic or anti-allogenic antibodies such as the anti-αGal, anti-A and anti-B antibodies, humans possess a large repertoire of natural anti-carbohydrate antibodies (New et al., 2016). Although most of them recognize bacterial structures, some have the potential to recognize viral glycans. Glycan structural analyses of the SARS-CoV-2 S protein produced in HEK-293T cells or of the virus produced in Vero cells have recently been described. They mainly include *N*-glycans of the oligomannose, hybrid and complex types that broadly cover the protein surface (Gao et al., 2020; Sanda et al., 2020; Shajahan et al., 2020; Sun et al., 2020; Watanabe et al., 2020). Yet, simple *O*-glycans have also been found either at the junction between the N-terminal domain (NTD) and the receptor binding domain (RBD) of the S1 domain or surrounding the furin cleavage site of the S2 domain (Antonopoulos et al., 2020; Gao et al., 2020; Sanda et al., 2020; Shajahan et al., 2020; Zhao P. et al., 2020). Based on these data, we selected a set of carbohydrate structures potentially present on virions produced by epithelial cells and known to constitute major epitopes of the human natural anti-carbohydrate repertoire (Huflejt et al., 2009; Stowell et al., 2014; Schneider et al., 2015; Muthana and Gildersleeve, 2016; Purohit et al., 2018; Khasbiullina et al., 2019).

Regardless of their specificity, levels of natural anti-glycans are highly variable between individuals (Tendulkar et al., 2017; Luetscher et al., 2020). Thus, we reasoned that if some natural anti-carbohydrate antibodies present an anti-viral activity, akin to what has been shown for anti-αGal antibodies in xenogenic situations (Galili, 2020), protection should not take place when they are present at low levels. Accordingly, patients should present lower levels of a protective anti-carbohydrate antibody specificity. In this work we thus compared levels of the selected anti-carbohydrate epitopes in the plasma of a group of COVID-19 patients and of a group of uninfected controls in order to reveal a potentially protective glycan epitope.

## Materials and Methods

### Study Design and Patients

For this study, the recruited individuals represented a subset from a study approved by the ethics committees of the Centre Hospitalier Universitaire Brugmann (CHU Brugmann, Bruxelles) and the Hôpital Universitaire Des Enfants Reine Fabiola (HUDERF, Bruxelles) in Belgium (the number “CHUB-BDS-Covid19 ClinicalTrials.gov: NCT04462627”). The study was carried out in accordance with the principles of the Declaration of Helsinki. The authors assume responsibility for the accuracy and completeness of the data and analyses.

Briefly, the study carried out between 11 March 2020 and 16 June 2020 in Brussels, Belgium, at the Brugmann University Hospital and the HUDERF, enabled the recruitment of 290 patients with or without symptoms of COVID 19, and with a positive RT-PCR test for SARS-CoV-2 on nasal and pharyngeal swab specimens. A control group (*n* = 276) included asymptomatic ambulatory patients or hospitalized patients without COVID symptoms and a negative RT-PCR test for SARS-CoV-2. For all these individuals, blood samples on EDTA were obtained on which standard anti-A and/or anti-B IgM agglutination scores were performed.

As shown on Table 1, to test our hypothesis about the involvement of other natural carbohydrate antibodies other than A and B in SARS-Cov-2 susceptibility, a random selection of 30 individuals from each of the patients and control groups A, B, and O types was performed (total target number = 180). The lack of left-over plasma from 10 selected individuals reduced our study sample to 170.

**TABLE 1:** Constitution of the controls and cases groups.

	Controls				Cases				
		
	A	B	O	Total controls	A	B	O	Total cases	Total samples
Complete study	108	52	116	276	126	48	116	290	566
Sub-study randomization	30	30	30	90	30	30	30	90	180
Tested samples	30	30	29	89	29	22	30	81	170
Actual sub study^*a*^	28 (−2)	28 (−2)	26 (−3)	82	31 (29 + 2)	24 (22 + 2)	33 (30 + 3)	88^*b*^	170

*^*a*^Seven controls were reclassified as cases because of anti-SARS-CoV-2 seropositivity.^*b*^Among cases, 75 were symptomatic and 13 were asymptomatic.*

Quantification of anti-SARS-CoV-2 antibodies (see method below) on the plasmas of individuals in the control group (with no apparent sign of COVID at the time of sampling and negative RT-PCR) showed that seven control individuals had antibodies indicating that they had been infected. For our analysis, these individuals were thus repositioned according to their ABO blood type in the patients’ group.

The patients’ group (88) was then subdivided into 2 subgroups: 75 COVID patients (RT-PCR positive and symptomatic), and 13 asymptomatic patients (RT-PCR positive or RT-PCR negative with positive serology resulting from the reclassification of the control individuals).

### Quantification of Natural Anti-carbohydrate Antibodies

**FIGURE 1:**
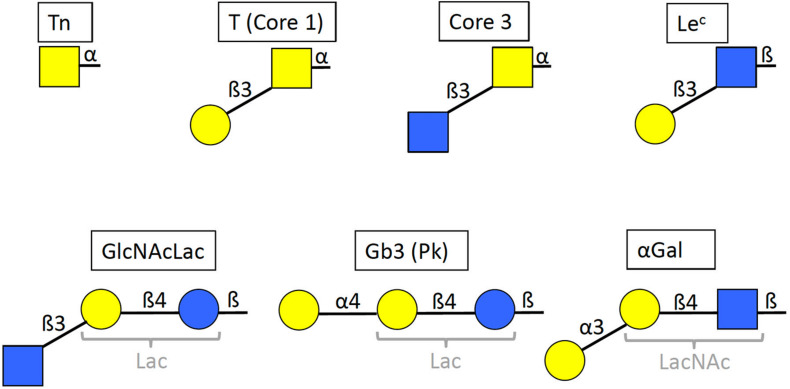
Structures of the selected glycan motifs. The Tn, T or core 1 and core 3 motifs correspond to short *O*-glycans in alpha linkage to either serine or threonine of the peptide chain. The Le^*c*^ (Lewis c), GlcNAcLac and αGal epitopes can be present either on N– and O–linked glycans of glycoproteins, or on glycolipids, whilst the Gb3 trisaccharide (globotrihexosyl), also called Pk antigen, is only known on glycosphingolipids. The GlcNAcLac, Gb3 and αGal motifs contain either a lactose or an *N*-acetyllactosamine inner core (Lac and LacNAc, respectively, in gray). Yellow squares = GalNAc (*N*-acetylgalactosamine), yellow circles = Gal (galactose), blue squares = GlcNAc (N-acetylglucosamine), blue circles = Glc (glucose). Linkages are indicated as α or β anomers on either position 3 or 4 of the subjacent monosaccharide unit.

Anti-carbohydrate antibodies were assessed with the enzyme-linked immunosorbent assay (ELISA). ELISA plates (Maxisorp, Nunc, Thermo Fisher Scientific, Roskilde, Denmark) were coated with synthetic sugars (structures shown on Figure 1) conjugated to polyacrylamide (PAA neoglycoconjugates) at 5 μg/mL in 0.1 M Carbonate buffer pH 9.0 overnight at 4°C. The plates were washed three times with phosphate-buffered saline (PBS)−0.05% Tween 20 (PBS-T), and unbound sites were blocked with PBS-5% bovine serum albumin (BSA) for 2 h at 37°C. After three additional washes with PBS-T, plasma samples (EDTA) from patients with Covid-19 or controls were added to the plate at a 1:30 dilution in PBS-1% BSA for 1 h at 37°C, except in the case of anti-αGal where plasma samples were diluted 1:50. Optimal dilutions had been chosen based on preliminary analyses performed using plasma samples from healthy blood donors. The plates were then washed 3 times with PBS-T and Donkey anti-human IgG (H + L)-conjugated horseradish peroxidase (Jackson ImmunoResearch Laboratories Inc., Ely, United Kingdom) was added at a 1:5000 dilution in PBS-1% BSA for 1 h at 37°C. This secondary antibody recognizes all classes of immunoglobulins, including IgM, IgG and IgA. Finally, after three last washes with PBS-T and one with plain PBS, revelation was performed with 50 μL/well of 3,3′,5,5′-Tetramethylbenzidine (Sigma Aldrich, St Louis, MO, United States) and the reaction was stopped with 50 μL/well of 1 M Phosphorous acid. Optical densities were read twice at 450 nm with a SPECTROstar Nano spectrophotometer (BMG Labtech, Champigny-sur-Marne, France).

### Flow Cytometry Quantification of Anti-SARS-CoV-2 Antibodies

The S-flow assay described by Grzelak et al. (2020) was used to detect anti-S viral protein in both controls and patients plasma samples. Briefly, detached HEK-293T cells (5 × 10^5^) stably expressing the S protein following transfection of a codon-optimized SARS-CoV-2 S gene were incubated for 30 min at 4°C with 50 μL of plasma samples at a 1/300 dilution in PBS containing 2 mM EDTA and 0.5% BSA. Untransfected HEK-293T cells used as negative controls were treated similarly. Following washings with PBS, cells were then incubated 30 min at 4°C with 35 μL of anti-Hu IgG (H + L) AlexaFluor 647 antibody (A21445, Invitrogen) diluted 1:600 in Staining Buffer. After washings in PBS, cells were fixed with PFA 2% (15714, Electron Microscopy Sciences, Hatfield, PA, United States), 15 min at room temperature, washed in PBS and analyzed by Flow cytometry, on a FACS Canto cytometer. Results were normalized according the formula: % of positive cells = 100 × [(% in 293T-S)−(% in 293T-CTRL)/100−(% in 293T-CTRL)]. The cut-off for positivity was fixed at 30%.

### Specificity Assay for the Anti-Tn NAM217-2A9 by ELISA

Maxisorp ELISA plates were coated with PAA-conjugated glycans at 10 μg/ml, human salivary mucins at 1:1000 dilution or bovine submaxillary mucins (Sigma) at 5 μg/ml in PBS at 4°C overnight. The sialyl-Tn-rich bovine mucins had been chemically de-sialylated by incubation in 2M H_2_SO_4_ for 30 min at 80°C followed by neutralization with NaOH. After three washes with PBS-0,05% Tween20 (PBS-T), the wells were blocked with PBS-5% BSA for 2 h at 37°C and washed another three times with PBS-T. Three-fold serial dilutions (1:50 to 1:1350) of mouse anti-Tn NAM217-2A9 in PBS-1%BSA were then loaded onto the plate and incubated for 1 h at 37°C. Washes, incubation with anti-mouse-HRP (Uptima, Interchim, Montluçon, France; 1:1000), revelation and OD measurement were then performed as described above.

#### Flow Cytometry Detection of the Tn Antigen

Vero green monkey kidney cells and HEK293T human embryonic kidney cells were detached using trypsin or PBS-EDTA, respectively, and resuspended in PBS-0.1% BSA. Jurkat cells were cultivated in suspension. 250.000 cells were stained with anti-Tn monoclonal mouse antibodies for 30–60 min at 4°C followed by anti-mouse-FITC 1:200. Analysis was performed on a Celesta flow cytometer using the DIVA software (BD Biosciences).

#### Immunohistological Analysis of the Expression of the Tn Antigen in the Respiratory Tract

The ethanol-fixed human tissue sections, collected and stored before the French law 88–138 of the 20th of December 1988 on tissue resection for scientific investigation, were obtained from the Nantes University Hospital Center for Biological Resources^1^ (approval DC-2011-1399).

Immunohistochemistry was performed as described elsewhere (Lopes et al., 2018). Briefly, the slides were deparaffinized and blocked for endogenous peroxidase activity and non-specific protein binding and incubated with the IgM mouse monoclonal antibodies against Tn NaM217-2A9 that was raised against human Tn erythrocytes (Duk et al., 2001) overnight at 4°C. The slides were then successively incubated with HRP-conjugated anti-mouse IgG (H + L) (Uptima; Interchim, Montluçon, France) and AEC substrate (Vector Laboratories, Burlingame, CA, United States) with three PBS washes in-between and conterstained with hematoxylin (Vector Laboratories) before mounting and imaging with a Nanozoomer slide-scanner using a ×20 objective (Hamamatsu Photonics, Massy, France).

## Statistical Analyses

Analyses were performed using GraphPad Prism 8. Between groups differences were calculated using two-tailed Mann-Whitney test and correlation were assessed using Pearson correlation coefficient. To account for multiple testing, Holm correction was applied were necessary. *P* < 0.05 was considered significant.

## Results

### Low Levels of Natural Anti-Tn Are Associated With COVID-19 Status

In order to select carbohydrate epitopes that may be present on SARS-CoV-2 and correspond to epitopes of the human natural anti-carbohydrate repertoire, we first examined published glycan microarray data that describe this repertoire (Huflejt et al., 2009; Stowell et al., 2014; Schneider et al., 2015; Muthana and Gildersleeve, 2016; Purohit et al., 2018; Khasbiullina et al., 2019). We looked for epitopes that could both be present on SARS-CoV-2 and give strong IgM and IgG signals with the serum of many healthy donors. The analysis revealed six potentially interesting epitopes, the short *O*-glycans Tn, T and core 3 as well as the Le^*c*^ and GlcNAcLac motifs of either complex *O*-glycans or *N*-glycans that can be synthesized by upper respiratory tract cells (Figure 1). In addition, we selected the Gb3 trisaccharide which corresponds to a widely distributed glycolipid highly reactive with healthy human serum natural antibodies (Volynsky et al., 2017).

**FIGURE 2:**
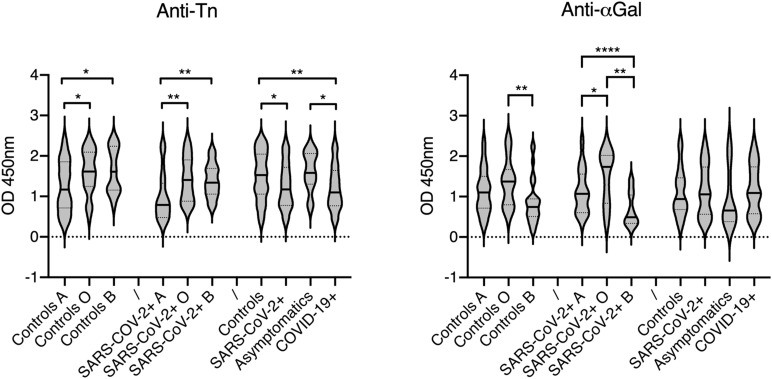
Relationships between the anti-Tn and anti-αGal levels, ABO phenotypes and infection status. Antibodies levels are shown as OD450 nm values at 1: 30 and 1: 50 plasma dilutions, respectively. Infected patients are defined as SARS-CoV-2 +, regardless of their clinical status or subdivided as asymptomatic and symptomatic COVID-19 +. Controls presented no sign of disease, were RT-PCR negative and anti-S negative. Violin plots show median values and quartiles (horizontal bars) for each group. *P*-values from Mann-Whitney between-groups comparisons are indicated: *p < 0.05, **p < 0.01, ****p < 0.0001.

**FIGURE 3:**
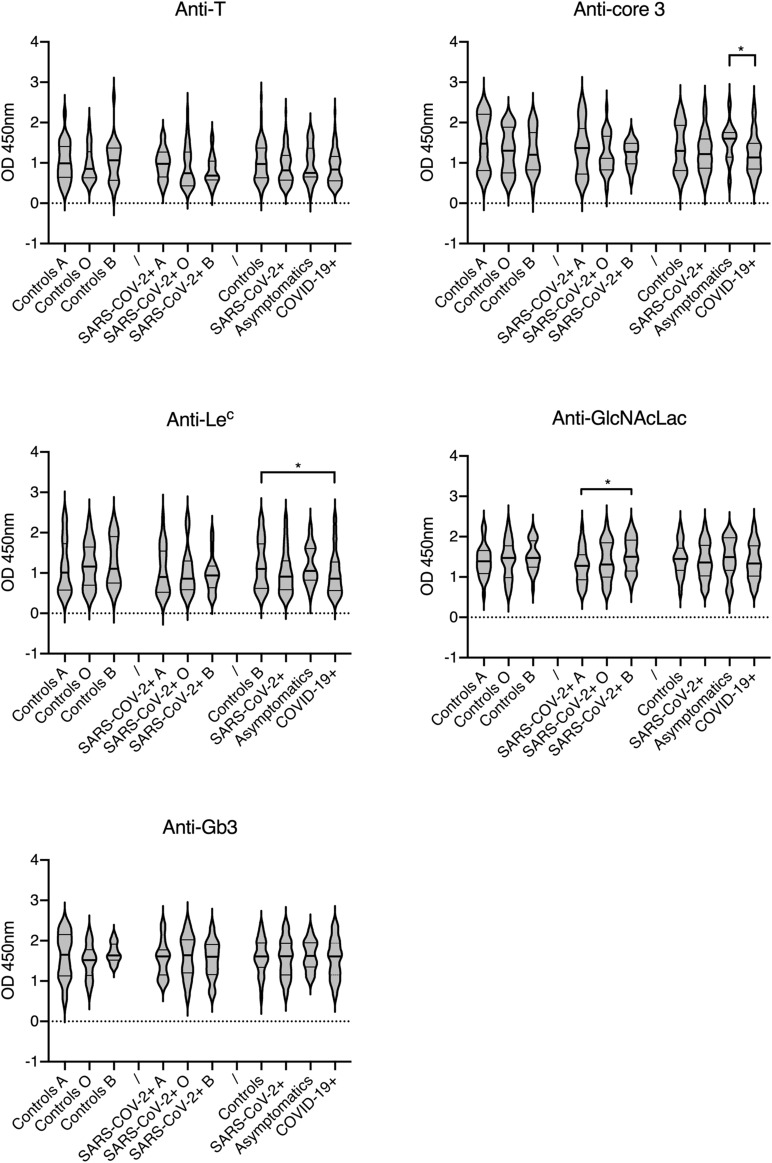
Relationships between natural anti-carbohydrate antibodies, ABO phenotypes and infection status. Analyzed carbohydrate antigens are indicated on each panel. Antibodies levels are shown as OD450 nm values at 1: 30 plasma dilutions. Infected patients are defined as SARS-CoV-2 +, regardless of their clinical status or subdivided as asymptomatic and symptomatic COVID-19 +. Controls presented no sign of disease, were RT-PCR negative and anti-S negative. Plots show median values and quartiles (horizontal bars). *P*-values from Mann-Whitney between-groups comparisons are indicated: *p < 0.05.

Individual levels of the selected natural anti-carbohydrate antibodies were then tested in a group of COVID-19 patients and compared to those in a group of controls of similar size. Since some of the tested antigens show similarity with either the A or B blood group antigens, the patients and controls groups were constituted so as to comprise nearly even numbers of A, B and O phenotypes. The αGal antigen was used as a control since it is not expressed in humans. We therefore anticipated that anti-αGal antibodies do not play any direct role in COVID-19 infection. Considering the structural relationship with the A blood group antigen, levels of anti-Tn were expected to be lower in blood group A individuals in comparison with blood group O and B and this was verified both for the control and COVID-19 groups (Figure 2). Interestingly, anti-Tn levels were significantly lower in COVID-19 patients as compared with controls since patients’ values were mainly distributed in the low range (*p* = 0.003). A similar, albeit less prominent effect was also visible when comparing SARS-CoV-2 infected individuals and controls (*p* = 0.012). Yet, no difference between controls and the subgroup of asymptomatic infected individuals was visible. It should be stressed, however, that the latter comprises 13 individuals only. The anti-αGal antibodies revealed a distinct picture. They appeared at lower levels in blood group B than in blood groups A and O individuals, both in the controls and SARS-CoV-2 infected groups. This was expected since the αGal epitope is closely related to blood group B. Yet, there was no difference related to the SARS-CoV-2 infectious status or COVID-19 status. Analyzing anti-T, anti-core-3, anti-Le^*c*^, anti-GlcNAcLac and anti-Gb3 revealed either no or only marginal between-group differences that vanished when using a threshold of significance lower than 0.02 (Figure 3). Thus, anti-Tn levels appear to be specifically low in COVID-19 patients.

**FIGURE 4:**
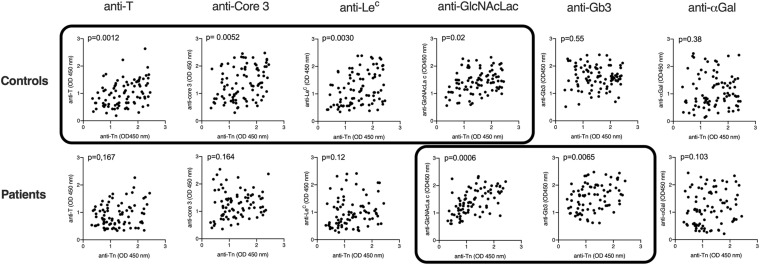
Correlations between levels of anti-Tn natural antibodies and the other assayed natural anti-carbohydrate antibodies. Antibodies levels are shown as OD450 nm values at 1: 30 or 1: 50 (αGal) plasma dilutions. Correlations were assessed using Spearman *r* and two-tailed *p*-values after Holm correction for multiple testing are shown. Data sets with *p*-values <0.05 are boxed.

Recently published data indicated that natural anti-glycan antibodies, including anti-Tn show cross-reactivity with a variety of other carbohydrate structures (Bovin et al., 2012; Dobrochaeva et al., 2020). Based on the premises that specific antibodies may cross-react with closely related structures, whilst less specific antibodies would cross-react more broadly, to get a more qualitative comparison of the natural antibodies from COVID-19 patients and controls, we performed correlation analyses of anti-Tn with the other tested anti-carbohydrates in both groups. In controls, strong correlations were found between anti-Tn levels and anti-T, anti-core 3 and anti-Le^*c*^ levels. A weaker correlation was additionally found with anti-GlcNAcLac. By contrast, in patients, anti-Tn strongly correlated with anti-GlcNAcLac and Gb3 only (Figure 4 and Table 2). These divergent relationships between levels of anti-Tn and those of the other anti-glycans in patients and controls indicate differences in natural antibodies repertoires between the two groups. Thus, not only do patients have lower levels of anti-Tn antibodies than controls, but these antibodies also appear qualitatively distinct.

**TABLE 2:** Correlations between levels of anti-Tn and levels of the other assayed natural anti-carbohydrates.

	Anti-T	Anti-core 3	Anti-Le^*c*^	Anti-GlcNAcLac	Anti-Gb3	Anti-αGal
						
Controls *N* = 82	0.38 (0.0002)	0.34 (0.0013)	0.36 (0.0006)	0.29 (0.0067)	−0.06 (0.5514)	0.09 (0.3826)
						
Patients *N* = 88	0.16 (0.1666)	0.16 (0.1644)	0.24 (0.0303)	0.58 (0.00001)	0.35 (0.0013)	0.18 (0.1032)

*Correlations were assessed using Spearman *r* and non-corrected for multiple testing two-tailed *p* values in parentheses are shown both for controls and patients. *R* values above 0.3 are boxed in red.*

### Correlation Between Anti-Tn and Anti-SARS-CoV-2 S Protein

**FIGURE 5:**
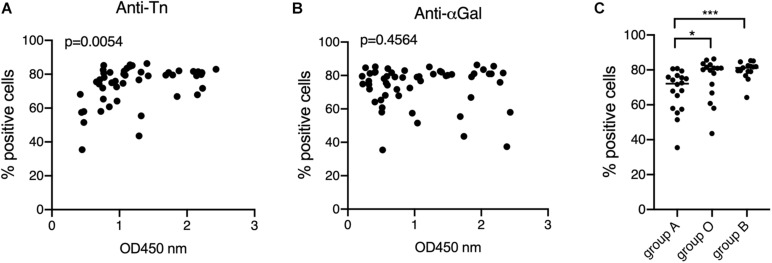
Relationships between the anti-Tn and anti-αGal levels, ABO phenotypes and the levels of anti-S protein. Anti-S protein antibodies of COVID-19 patients were detected and quantified by the S-Flow assay at a plasma dilution of 1: 300. Data are shown as percentage of positive cells. Only positive plasma samples were considered. **(A)** Correlation between anti-S and anti-Tn, Spearman *r p* value is shown; **(B)** correlation between anti-S and anti-αGal, Spearman *r P*-value is shown; **(C)** relationship between ABO phenotypes and anti-S. Only anti-S positive individuals (with cut-off values >30%) were considered. *P*-values from Mann-Whitney comparisons are indicated: *p < 0.05, ***p < 0.001.

Having observed that anti-Tn levels were lower in patients than in controls, we wondered if their levels within patients might have a relationship with the development of the specific anti-viral immune response. Anti-S protein antibodies were quantified using the previously described S-flow assay that shows high correlation with the neutralization assay (Grzelak et al., 2020). Anti-S antibodies were found in 45 out of 75 symptomatic patients, a rather low percentage (60%), likely resulting from the early sampling at the time of diagnosis of some patients. Interestingly, anti-S antibodies levels correlated with those of anti-Tn, unlike anti-αGal (Figures 5A,B) and the remaining tested anti-carbohydrates (not shown). Consistent with the lower levels of anti-Tn in blood group A individuals, it appeared that anti-S antibodies were also lower in blood group A individuals as compared to blood group O and blood group B patients (Figure 5C).

### Expression of the Tn Epitope in the Respiratory Tract

**FIGURE 6:**
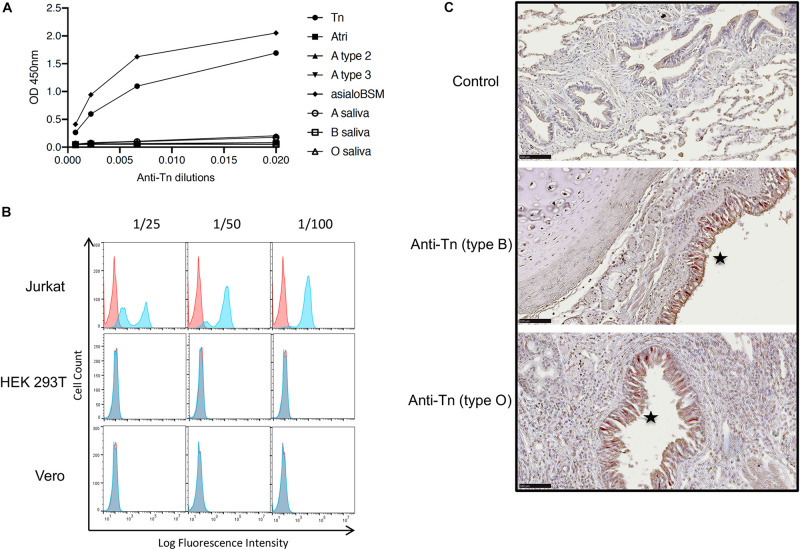
Expression of the Tn epitope in lung and trachea. **(A)** Binding of the anti-Tn monoclonal antibody NaM217-2A9 to immobilized polyacrylamide-conjugated carbohydrate epitopes (Tn, A blood group trisaccharide, A type 2 and A type 3), human salivary mucins from A, B or O secretor individuals and desialylated bovine submaxillary mucin (asialoBSM) were assayed by ELISA. Strong reactivity was observed with the Tn-PAA conjugate, and asialoBSM that contains large amounts of Tn epitope. **(B)** Reactivity of the anti-Tn on cell lines detected by flow cytometry. Jurkat cells known to express large amounts of Tn epitope were used as a positive control. Negative controls are shown as red plots the blue plots showing data in the presence of the anti-Tn NaM217-2A9. **(C)** Staining of tracheal (middle panel) and bronchial epithelial cells (lower panel) by the anti-Tn NaM217-2A9 shown by the brown-red color (stars) in a blood group B and a blood group O donor, respectively (type B, type O). The upper panel (negative control) shows the lack of staining in absence of the primary anti-Tn antibody. Black bar = 100 μm.

The Tn antigen has been detected on SARS-CoV-2 S protein produced in HEK-293T cells (Gao et al., 2020; Sanda et al., 2020; Shajahan et al., 2020). Their *O*-glycosylation capability unlikely represents that of epithelial cells of the respiratory tract which are the main viral target cells contributing to transmission. Respiratory as well as digestive epithelial cells produce large amounts of *O*-glycans and express a broad set of the polypeptide GalNAc transferases that add the first *N*-acetylgalatosamine unit to the peptide chain constituting the Tn epitope (Bennett et al., 2012). In normal tissues, the Tn antigen is masked by elongation of *O*-glycan chains and it is known to be over-expressed in carcinoma (Rodrigues et al., 2018). Nonetheless, in order to determine if indeed the Tn antigen could be produced by respiratory epithelial cells, we tested its expression by immunohistochemistry on fixed tracheal and lung tissue from three individuals of the A, B and O blood types, respectively. First, the anti-Tn specificity was validated by ELISA on PAA neoglycoconjugates and on mucins presenting high levels of either the Tn epitope or A, B and H blood group antigens. We observed a specific binding to the Tn-PAA conjugate and to Tn-rich desialylated bovine submaxillary mucin, but not to other conjugates or to human salivary mucin, although a small reactivity was detected on blood group A containing mucin or neoglycoconjugate (Figure 6A). In addition, the antibody had previously been shown to recognize glycophorins from Tn erythrocytes, as well as ovine submaxillary mucin (OSM) (Duk et al., 2001). Thus, the anti-Tn that we used recognizes the Tn antigen independently of the underlying peptide while showing only little cross-reactivity with related structures such as blood group A antigen. Flow cytometry experiments additionally showed that the anti-Tn strongly bound to Jurkat cells known to strongly express the Tn epitope due to a genetic defect that impairs *O*-glycans elongation. In the same conditions, HEK-293T cells and Vero cells were not recognized by the antibody (Figure 6B). When tested on tissue sections, the antibody revealed a strong intracellular binding to the tracheal and bronchial epithelia, but not to any other cell type in the respiratory tract, regardless of the donor ABO phenotype (Figure 6C).

## Discussion

Here we tested the possibility that in addition to anti-A and anti-B blood group antibodies, natural anti-carbohydrate antibodies could play a protective role against SARS-CoV-2 infection. Consistent with the initial hypothesis, we observed that COVID-19 patients presented lower levels of anti-Tn antibodies than controls, whilst none of the other tested anti-carbohydrates showed significant differences in levels between the two groups. Tn antigen is constituted by a single *N*-acetylgalactosamine linked to either a serine or a threonine residue. It constitutes the basis of all mucin-type *O*-glycans to which additional monosaccharide units are generally added. It is therefore not normally detected at the cell surface and is considered a tumor marker in several types of carcinoma where elongation of *O*-glycosylation is impaired (Rodrigues et al., 2018). It is also considered a blood group isoantigen since there exist rare individuals who express the Tn antigen on their erythrocytes due to a genetic defect in the *O*-glycosylation elongation pathway and since natural anti-Tn antibodies agglutinate these rare erythrocytes, causing a so-called polyagglutinability (Dahr et al., 1975).

The SARS-CoV-2 S protein possesses several documented *O*-glycosylation sites located at the hinge between the RBD and NTD domains and surrounding the furin cleavage site (Antonopoulos et al., 2020; Gao et al., 2020; Sanda et al., 2020; Shajahan et al., 2020; Zhao P. et al., 2020). Blocking of *O*-glycosylation resulted in partial inhibition of SARS-CoV-2 cell entry *in vitro*, indicating the functional importance of *O*-glycans in the infection process (Yang et al., 2020). Epithelial cells of the upper respiratory tract, nasopharynx, trachea and large bronchi, appear to be the main producers of virions involved in inter-individual transmission (Wolfel et al., 2020). In accordance with the notion that epithelial cells synthesize large amounts of *O*-glycans, we observed that these cells in the respiratory epithelia present intracellular Tn epitopes, unlike the remaining cell types present in these tissues. Initiation of GalNAc-type *O*-glycosylation is performed by polypeptide *N*-acetylgalatosaminyltransferases (GalNAc-Ts) (Bennett et al., 2012). Twenty isoforms of GalNAc-Ts are known that show both overlapping and non-redundant specific functions to orchestrate the patterns of *O*-glycans on proteins. Inspection of the Human Protein Atlas^2^ indicates that epithelial cells of the nasopharynx and of bronchi express strong to moderate amounts of at least 11 of these enzymes. This confirmed the strong potential for epithelial cells of the nasopharynx, trachea and bronchi to produce *O*-glycosylated glycoproteins. At present, only small amounts of *O*-glycans, including the Tn epitope, have been detected on SARS-CoV-2 recombinant S protein produced in HEK-293 cells, indicating that the major viral envelope protein can be *O*-glycosylated. Rapidly replicating viruses being expected to carry a large fraction of immature glycan structures, it is thus plausible that authentic infectious SARS-CoV-2 carries Tn epitopes.

Confirmation will await the complete structural characterization of native expectorated virions. In addition, it will be necessary in future experiments to show that purified natural anti-Tn antibodies can bind to native virions.

Since blood group A antigen is characterized by a terminal *N*-acetylgalactosamine in alpha linkage, it was expected that the levels of anti-Tn antibodies would be lower in blood group A individuals owing to the structural homology between the two epitopes. Likewise, due to the homology between blood group B and the αGal antigen, it was expected that blood group B individuals should show lower anti-αGal levels. These associations with ABO phenotypes were indeed observed for the Tn and αGal antigens, respectively, but not for any of the other tested carbohydrate epitopes, which validated the method of quantification that we used. Regarding analyses of correlations with COVID-19 status, only the anti-Tn antigen showed significant lower amounts in patients in comparison with the control group, indicating that the effect did not stem from a general decrease of natural anti-carbohydrate in patients following infection. Thus, the data suggest that patients had lower anti-Tn prior to being infected. However, at this stage we cannot eliminate other potential causes that might contribute to the difference in anti-Tn levels between patients and controls. Besides the quantitative difference, there was a qualitative difference between patients and controls anti-Tn antibodies. The qualitative difference was indirectly observed through analysis of correlations with the other carbohydrate epitopes, revealing an absence of correlation with Tn-related motifs for patients, unlike for controls. A previous study performed using purified anti-Tn antibodies from pooled plasma samples revealed cross-reactivities with several related oligosaccharides or unrelated polysaccharides, indicating a rather broad polyreactivity (Bovin et al., 2012; Dobrochaeva et al., 2020). It is possible that the divergent patterns of correlation with other anti-carbohydrates between patients and control groups reflect distinct cross-reactivities of the anti-Tn. Alternatively, the observed divergent correlations between patients and controls for several anti-carbohydrates could reflect divergent immunogens composition from the microbiota. Direct evidence for this could be obtained by a specificity analysis following purification of patients and controls anti-Tn antibodies in future studies. In addition, it will be important to distinguish between different immunoglobulin subclasses, which was not done in this preliminary study.

Levels of natural anti-carbohydrate antibodies may decrease with aging (Muthana and Gildersleeve, 2016). Since patients and controls groups were not matched for age, the lower anti-Tn observed in patients may have originated from the higher mean age of patients (68 years) compared to that of controls (42 years). Again, this seems unlikely since the same effect should have been observed for the other anti-carbohydrates that were tested. In addition, within the patients’ cohort there was a relationship between the levels of anti-Tn, but not of the other tested anti-carbohydrate antibodies, and those of anti-S viral protein. Although the underlying reason for this correlation remains unknown, it is noteworthy that similar to anti-Tn antibodies, the anti-S antibodies were lower in blood group A patients, suggesting that anti-Tn may have contributed to the development of the specific anti-viral response, akin to what has previously been observed for the anti-αGal antibodies in models of xenogenic viral infections (Galili, 2020).

It is now rather well documented through a large set of studies that blood group A individuals are at a higher risk of COVID-19 than individuals of blood group O, whilst blood group B seldom shows significant odd ratios relative to the other blood groups (Aktimur et al., 2020; Barnkob et al., 2020; Chegni et al., 2020; Ellinghaus et al., 2020; Göker et al., 2020; Latz et al., 2020; Leaf et al., 2020; Li et al., 2020; Muniz-Diaz et al., 2020; Shelton et al., 2020; Sohlpour et al., 2020; Valenti et al., 2020; Wu et al., 2020; Zeng et al., 2020; Zhang et al., 2020; Zhao J. et al., 2020; Zietz et al., 2020). We recently observed that anti-A and anti-B agglutinating natural antibodies were significantly lower in COVID-19 patients compared with controls (Deleers et al., 2020). Together with the present observations on anti-Tn antibodies, this may explain the ABO effect on COVID-19 epidemiology. Indeed, blood group O individuals, possess natural anti-A, anti-B and anti-Tn antibodies, B blood group individuals possess anti-A and anti-Tn antibodies, but blood group A individuals possess anti-B and low levels of anti-Tn only. The degree of protection conferred by these natural anti-carbohydrates would thus be minimal for blood group AB individuals who should possess only low levels of anti-Tn, followed by blood group A, then blood group B and finally by blood group O individuals who could benefit from all three types of antibodies. The anti-Tn antibodies could be particularly important as they could provide protection regardless of the ABO type of the virus transmitters, unlike anti-A and anti-B that may only protect during transmission events in an ABO incompatible situation.

In conclusion, in a first exploratory study, we observed that natural anti-Tn antibodies differ between COVID-19 patients and controls both quantitatively and qualitatively and that their levels are lower in blood group A individuals and associated with the levels of anti-S protein. These results suggest that natural anti-carbohydrate antibodies that target *O*-glycans may confer some protection against COVID-19. Nonetheless, it should be stressed that our study is preliminary and requires validation by further studies including age and gender matching of patients and controls, as well as *in vitro* experiments aimed at characterizing the mechanisms whereby anti-Tn antibodies could be protective. If validated, it would indicate that a significant fraction of the population with sufficient natural anti-Tn antibodies could benefit from a natural immunity conferred by these antibodies against COVID-19 and it would offer a prophylactic perspective by boosting the anti-Tn titers in everyone.

## Data Availability Statement

The raw data supporting the conclusions of this article will be made available by the authors, without undue reservation.

## Ethics Statement

The studies involving human participants were reviewed and approved by Centre Hospitalier Universitaire Brugmann (CHU Brugmann, Bruxelles) and the Hôpital Universitaire Des Enfants Reine Fabiola (HUDERF, Bruxelles). The patients/participants provided their written informed consent to participate in this study.

## Author Contributions

AB, NR-C, TB, NJ, and JR performed the experiments. MD and HE provided the patients samples, their ABO blood groups and COVID-19 status. NB provided reagents and discussed the data. NL, AB, and NRV analyzed the data. JLP conceived the work, analyzed the data and wrote the manuscript. All authors corrected the manuscript and approved the final version.

## Conflict of Interest

The authors declare that the research was conducted in the absence of any commercial or financial relationships that could be construed as a potential conflict of interest.

## Footnotes

1 http://relib.fr

2 www.proteinatlas.org

## References

[B1] Abdollahi A. Mahmoudi-Aliabadi M. Mehrtash V. Jafarzadeh B. Salehi M. (2020). The novel coronavirus SARS-CoV-2 vulnerabiloty association with ABO/Rh blood types. *Iran. J. Pathol.* 15 156–160. 10.30699/ijp.2020.125135.2367 32754209 PMC7354076

[B2] Ahmed I. Quinn L. Tan B. K. (2020). COVID-19 and the ABO blood group in pregnancy: a tale of two multiethnic cities. *Int. J. Lab. Hematol.* 43, e45–e47. 10.1111/ijlh.13355 32996710 PMC7537203

[B3] Aktimur S. H. Sen H. Yazicioglu B. Gunes A. K. Genc S. (2020). The assessment of the relationship between ABO blood groups and Covid-19 infection. *Int. J. Hematol. Oncol.* 30 121–125. 10.4999/uhod.204348

[B4] Aljanobi G. Alhajjaj A. Alkhabbaz F. Al-Jishi J. (2020). The relationship between ABO blood group type and the covid-19 susceptibility in qatif central hospital, eastern province, saudi arabia: a retrospective cohort study. *Open J. Intern. Med.* 10 232–238. 10.4236/ojim.2020.102024

[B5] Antonopoulos A. Broome S. Sharov V. Ziegenfuss C. Easton R. L. Panico M. (2020). Site-specific characterisation of SARS-CoV-2 spike glycoprotein receptor binding domain. *Glycobiology* 10.1093/glycob/cwaa085 32886791 PMC7499654

[B6] Bagdonaite I. Wandall H. H. (2018). Global aspects of viral glycosylation. *Glycobiology* 28 443–467. 10.1093/glycob/cwy021 29579213 PMC7108637

[B7] Barnkob M. B. Pottegard A. Stovring H. Haunstrup T. M. Homburg K. Larsen R. (2020). Reduced prevalence of SARS-CoV-2 infection in ABO blood group O. *Blood Adv.* 4 4990–4993. 10.1182/bloodadvances.2020002657 33057631 PMC7594382

[B8] Bennett E. P. Mandel U. Clausen H. Gerken T. A. Fritz T. A. Tabak L. A. (2012). Control of mucin-type O-glycosylation: a classification of the polypeptide GalNAc-transferase gene family. *Glycobiology* 22 736–756. 10.1093/glycob/cwr182 22183981 PMC3409716

[B9] Boudin L. Janvier F. Bylicki O. Dutasta F. (2020). ABO blood groups are not associated with risk of acquiring the SARS-CoV-2 infection in young adults. *Haematologica* 105 2841–2843. 10.3324/haematol.2020.265066 33256383 PMC7716357

[B10] Bovin N. Obukhova P. Shilova N. Rapoport E. Popova I. Navakouski M. (2012). Repertoire of human natural anti-glycan immunoglobulins. do we have auto-antibodies? *Biochim. Biophys. Acta* 1820 1373–1382. 10.1016/j.bbagen.2012.02.005 22365885

[B11] Chegni H. Pakravan N. Saadati M. Ghaffari A. D. Shirzad H. Hassan Z. M. (2020). Is There a link between COVID-19 mortality with genus, age, ABO blood group type, and ACE2 gene polymorphism? *Iran. J. Public Health* 49 1582–1584. 33083341 10.18502/ijph.v49i8.3910 PMC7554399

[B12] Cheng Y. Cheng G. Chui C. H. Lau F. Y. (2005). ABO blood group and susceptibility to severe acute respiratory syndrome. *JAMA* 293 1450–1451. 10.1001/jama.293.12.1450-c 15784866

[B13] Dahr W. Uhlenbruck G. Gunson H. H. Van Der Hart M. (1975). Molecular basis of Tn-polyaggutinability. *Vox. Sang.* 29 36–50. 10.1111/j.1423-0410.1975.tb00475.x 1173700

[B14] Delanghe J. R. De Buyzere M. L. Speeckaert M. M. (2020). C3 and ACE1 polymorphisms are more important confounders in the spread and outcome of COVID-19 in comparison with ABO polymorphism. *Eur. J. Prev. Cardiol.* 27 1331–1332. 10.1177/2047487320931305 32460534 PMC7717311

[B15] Deleers M. Breiman A. Daubie V. Maggetto C. Barreau I. Besse T. (2020). Covid-19 and blood groups: ABO antibody levels may also matter. *Int. J. Infect. Dis.* 104, 242–249. 10.1016/j.ijid.2020.12.025 33326874 PMC7832075

[B16] Dobrochaeva K. Khasbiullina N. Shilova N. Antipova N. Obukhova P. Ovchinnikova T. (2020). Specificity of human natural antibodies referred to as anti-Tn. *Mol. Immunol.* 120 74–82. 10.1016/j.molimm.2020.02.005 32087569

[B17] Duk M. Blanchard D. Lisowska E. (2001). Anti-T and anti-Tn antibodies. *Trans. Clin. Biol.* 8 253.

[B18] Dzik S. Eliason K. Morris E. B. Kaufman R. M. North C. M. (2020). COVID-19 and ABO blood groups. *Transfusion* 60 1883–1884. 32562280 10.1111/trf.15946 PMC7323215

[B19] Ellinghaus D. Degenhardt F. Bujanda L. Buti M. Albillos A. Invernizzi P. (2020). Genomewide association study of severe covid-19 with respiratory failure. *N. Engl. J. Med.* 383 1522–1534. 10.1056/nejmoa2020283 32558485 PMC7315890

[B20] Fan Q. Zhang W. Li B. Li D. J. Zhang J. Zhao F. (2020). Association between ABO blood group system and COVID-19 susceptibility in Wuhan. *Front. Cell Infect. Microbiol.* 10:404. 10.3389/fcimb.2020.00404 PMC7385064 32793517

[B21] Focosi D. Iorio M. C. Lanza M. (2020). ABO blood group correlations with COVID-19: cohort choice makes a difference. *Clin. Infect. Dis.* 361:ciaa1495. 10.1093/cid/ciaa1495 32997752 PMC7543376

[B22] Franchini M. Glingani C. Del Fante C. Capuzzo M. Di Stasi V. Rastrelli G. (2020). The protective effect of O blood type against SARS-CoV-2 infection. *Vox Sang* (in press). 10.1111/vox.13003 32950039 PMC7537255

[B23] Galili U. (2019). Evolution in primates by “Catastrophic-selection” interplay between enveloped virus epidemics, mutated genes of enzymes synthesizing carbohydrate antigens, and natural anticarbohydrate antibodies. *Am. J. Phys. Anthropol.* 168 352–363. 10.1002/ajpa.23745 30578545

[B24] Galili U. (2020). Human natural antibodies to mammalian carbohydrate antigens as unsung heroes protecting against past, present, and future viral infections. *Antibodies (Basel)* 9:25 10.3390/antib9020025 PMC7344964 32580274

[B25] Gallian P. Pastorino B. Morel P. Chiaroni J. Ninove L. Lamballerie X. (2020). Lower prevalence of antibodies neutralizing SARS-CoV-2 in group O French blood donors. *Antiviral. Res.* 181:104880. 10.1016/j.antiviral.2020.104880 32679056 PMC7362788

[B26] Gao C. Zeng J. Jia N. Stavenhagen K. Matsumoto Y. Zhang H. (2020). SARS-CoV-2 spike protein interacts with multiple innate immune receptors. *bioRxiv* [Preprint]. 10.1101/2020.1107.1129.227462

[B27] Göker H. Aladağ K. E. Demiroğlu H. Ayaz Ceylan ÇM. Büyükaşik Y. Inkaya A. Ç, et al. (2020). The effects of blood group types on the risk of COVID-19 infection and its clinical outcome. *Turk. J. Med. Sci.* 50 679–683. 10.3906/sag-2005-395 32496734 PMC7379446

[B28] Grzelak L. Temmam S. Planchais C. Demeret C. Tondeur L. Huon C. (2020). A comparison of four serological assays for detecting anti-SARS-CoV-2 antibodies in human serum samples from different populations. *Sci. Transl. Med.* 12:3103. 10.1126/scitranslmed.abc3103 32817357 PMC7665313

[B29] Guillon P. Clément M. Sébille V. Rivain J.-G. Chou C.-F. Ruvoën-Clouet N. (2008). Inhibition of the interaction beteen the SARS-CoV spike protein and its cellular receptor by anti-histo-blood group antibodies. *Glycobiology* 18 1085–1093. 10.1093/glycob/cwn093 18818423 PMC7108609

[B30] Hoiland R. L. Fergusson N. A. Mitra A. R. Griesdale D. E. G. Devine D. V. Stukas S. (2020). The association of ABO blood group with indices of disease severity and multiorgan dysfunction in COVID-19. *Blood Adv.* 4 4981–4989. 10.1182/bloodadvances.2020002623 33057633 PMC7594392

[B31] Huflejt M. E. Vuskovic M. Vasiliu D. Xu H. Obukhova P. Shilova N. (2009). Anti-carbohydrate antibodies of normal sera: findings, surprises and challenges. *Mol. Immunol.* 46 3037–3049. 10.1016/j.molimm.2009.06.010 19608278

[B32] Khasbiullina N. R. Shilova N. V. Navakouski M. J. Nokel A. Y. Blixt O. Kononov L. O. (2019). The repertoire of human antiglycan antibodies and its dynamics in the first year of life. *Biochemistry (Mosc)* 84 608–616. 10.1134/s0006297919060038 31238860

[B33] Latz C. A. Decarlo C. Boitano L. Png C. Y. M. Patell R. Conrad M. F. (2020). Blood type and outcomes in patients with COVID-19. *Ann. Hematol.* 99 2113–2118. 32656591 10.1007/s00277-020-04169-1 PMC7354354

[B34] Leaf R. K. Al-Samkari H. Brenner S. K. Gupta S. Leaf D. E. (2020). ABO phenotype and death in critically ill patients with COVID-19. *Br. J. Haematol.* 190, e204–e208. 10.1111/bjh.16984 32609874 PMC7361419

[B35] Li J. Wang X. Chen J. Cai Y. Deng A. Yang M. (2020). Association between ABO blood groups and risk of SARS-CoV-2 pneumonia. *Br. J. Haematol.* 190 24–27. 10.1111/bjh.16797 32379894 PMC7267665

[B36] Lopes A. M. Breiman A. Lora M. Le Moullac-Vaidye B. Galanina O. Nystrom K. (2018). Host-specific glycans are correlated with susceptibility to infection by lagoviruses, but not with their virulence. *J. Virol.* 92:e001759-17. 10.1128/JVI.01759-17 PMC5790946 29187537

[B37] Luetscher R. N. D. Mckitrick T. R. Gao C. Mehta A. Y. Mcquillan A. M. Kardish R. (2020). Unique repertoire of anti-carbohydrate antibodies in individual human serum. *Sci. Rep.* 10:15436. 10.1038/s41598-020-71967-y PMC7509809 32963315

[B38] Muniz-Diaz E. Llopis J. Parra R. Roig I. Ferrer G. Grifols J. (2020). Relationship between the ABO blood group and COVID-19 susceptibility, severity and mortality in two cohorts of patients. *Blood Transfus* 11 462–465. 10.2450/2020.0256-2420 PMC7850930 33196417

[B39] Muthana S. M. Gildersleeve J. C. (2016). Factors affecting anti-glycan IgG and IgM repertoires in human serum. *Sci. Rep.* 6:19509. 10.1038/srep19509 PMC4726023 26781493

[B40] New J. S. King R. G. Kearney J. F. (2016). Manipulation of the glycan-specific natural antibody repertoire for immunotherapy. *Immunol. Rev.* 270 32–50. 10.1111/imr.12397 26864103 PMC4755354

[B41] Niles J. K. Karnes H. E. Dlott J. S. Kaufman H. W. (2020). Association of ABO/Rh with SARS-CoV-2 positivity: The role of race and ethnicity in a female cohort. *Am. J. Hematol.* 96, E23–E26. 10.1002/ajh.26019 33064308 PMC7675235

[B42] Padhi S. Suvankar S. Dash D. Panda V. K. Pati A. Panigrahi J. (2020). ABO blood group system is associated with COVID-19 mortality: an epidemiological investigation in the Indian population. *Transfus Clin. Biol.* 27 253–258. 10.1016/j.tracli.2020.08.009 32987167 PMC7518849

[B43] Pairo-Castineira E. Clohisey S. Klaric L. Bretherick A. Rawlik K. Parkinson N. (2020). Genetic mechanisms of critical illness in Covid-19. *MedRxiv* [Preprint]. 10.1101/2020.1109.1124.20200048 33307546

[B44] Preece A. F. Strahan K. M. Devitt J. Yamamoto F. F. Gustavson K. (2002). Expression of ABO or related antigenic carbohydrates on viral envelopes leads to neutralization in the presence of serum containing specific natural antibodies and complement. *Blood* 99 2477–2482. 10.1182/blood.v99.7.2477 11895782

[B45] Purohit S. Li T. Guan W. Song X. Song J. Tian Y. (2018). Multiplex glycan bead array for high throughput and high content analyses of glycan binding proteins. *Nat. Commun.* 9:258. 10.1038/s41467-017-02747-y PMC5772357 29343722

[B46] Ray J. G. Schull M. J. Vermeulen M. J. Park A. L. (2020). Association between ABO and Rh blood groups and SARS-CoV-2 infection or severe COVID-19 Illness. a population-based cohort study. *Ann. Int. Med.* (in press) 10.7326/M7320-4511 PMC7711653 33226859

[B47] Roberts G. H. L. Park D. S. Coignet M. V. Mccurdy S. R. Knight S. C. Partha R. (2020). AncestryDNA COVID-19 host genetic study I 1 dentifies three novel loci. *medRxiv* [Preprint]. 10.1101/2020.1110.1106.20205864

[B48] Rodrigues J. G. Balmana M. Macedo J. A. Pocas J. Fernandes A. De-Freitas-Junior J. C. M. (2018). Glycosylation in cancer: selected roles in tumour progression, immune modulation and metastasis. *Cell Immunol.* 333 46–57. 10.1016/j.cellimm.2018.03.007 29576316

[B49] Sanda M. Morrison L. Goldman R. (2020). N and O glycosylation of the SARS-CoV-2 spike protein. *bioRxiv* [Preprint]. 10.1101/2020.1107.1105.187344 PMC7805595 33406838

[B50] Schneider C. Smith D. F. Cummings R. D. Boligan K. F. Hamilton R. G. Bochner B. S. (2015). The human IgG anti-carbohydrate repertoire exhibits a universal architecture and contains specificity for microbial attachment sites. *Sci. Transl. Med.* 7:269ra261. 10.1126/scitranslmed.3010524 PMC4864610 25568069

[B51] Shajahan A. Supekar N. T. Gleinich A. S. Azadi P. (2020). Deducing the N- and O- glycosylation profile of the spike protein of novel coronavirus SARS-CoV-2. *Glycobiology* 30, 981–988. 10.1093/glycob/cwaa042 32363391 PMC7239183

[B52] Shelton J. F. Shastri A. J. Ye C. Weldon C. H. Filshtein-Somnez T. Coker D. (2020). Trans-ethnic analysis reveals genetic and non-genetic associations with COVID-19 susceptibility and severity. *medRxiv* [Preprint]. 10.1101/2020.1109.1104.20188318 33888907

[B53] Sohlpour A. Jafari A. Apourhoseingholi M. A. Soltani F. (2020). Corona COVID-19 virus and severe hypoxia in young patients without underlying disease: high prevalence rate with blood group A. *Trends Anesth Crit. Care* 34 63–64. 10.1016/j.tacc.2020.1008.1005 PMC7428749 38620595

[B54] Stowell S. R. Arthur C. M. Mcbride R. Berger O. Razi N. Heimburg-Molinaro J. (2014). Microbial glycan microarrays define key features of host-microbial interactions. *Nat. Chem. Biol.* 10 470–476. 10.1038/nchembio.1525 24814672 PMC4158828

[B55] Sun Z. Ren K. Zhang X. Chen J. Jiang Z. Jiang J. (2020). Mass spectrometry analysis of newly emerging coronavirus HCoV-19 spike protein and human ACE2 reveals camouflaging glycans and unique post-translational modifications. *Engineering (Beijing)* (in press). 10.1016/j.eng.2020.1007.1014 PMC7456593 32904601

[B56] Tendulkar A. A. Jain P. A. Velaye S. (2017). Antibody titers in Group O platelet donors. *Asian J. Transfus. Sci.* 11 22–27. 10.4103/0973-6247.200765 28316436 PMC5345276

[B57] Valenti L. Villa S. Baselli G. Temporiti R. Bandera A. Scudeller L. (2020). Association of ABO blood groups and secretor phenotype with severe COVID-19. *Transfusion* 60, 3067–3070. 10.1111/trf.16130 33009662 PMC7675339

[B58] Volynsky P. Efremov R. Mikhalev I. Dobrochaeva K. Tuzikov A. Korchagina E. (2017). Why human anti-Galalpha1-4Galbeta1-4Glc natural antibodies do not recognize the trisaccharide on erythrocyte membrane? Molecular dynamics and immunochemical investigation. *Mol. Immunol.* 90 87–97. 10.1016/j.molimm.2017.06.247 28708979

[B59] Watanabe Y. Allen J. D. Wrapp D. Mclellan J. S. Crispin M. (2020). Site-specific glycan analysis of the SARS-CoV-2 spike. *Science* 369 330–333. 32366695 10.1126/science.abb9983 PMC7199903

[B60] Watanabe Y. Bowden T. A. Wilson I. A. Crispin M. (2019). Exploitation of glycosylation in enveloped virus pathobiology. *Biochim. Biophys. Acta Gen. Subj.* 1863 1480–1497. 10.1016/j.bbagen.2019.05.012 31121217 PMC6686077

[B61] Wolfel R. Corman V. M. Guggemos W. Seilmaier M. Zange S. Muller M. A. (2020). Virological assessment of hospitalized patients with COVID-2019. *Nature* 581 465–469. 10.1038/s41586-020-2196-x 32235945

[B62] Wu Y. Feng Z. Li P. Yu Q. (2020). Relationship between ABO blood group distribution and clinical characteristics in patients with COVID-19. *Clin. Chim. Acta* 509 220–223. 10.1016/j.cca.2020.06.026 32562665 PMC7832938

[B63] Yang Q. Hughes T. A. Kelkar A. Yu X. Cheng K. Park S. (2020). Inhibition of SARS-CoV-2 viral entry upon blocking N- and O-glycan elaboration. *Elife* 9:e61552. 10.7554/eLife.61552 PMC7685702 33103998

[B64] Zeng X. Fan H. Lu D. Meng F. Zhuo L. Tang M. (2020). Association between ABO blood groups and clinical outcome of coronavirus disease 2019: evidence from two cohorts. *medRxiv* [Preprint]. 10.1101/2020.1104.1115.20063107

[B65] Zhang L. Huang B. Xia H. Fan H. Zhu M. Zhu L. (2020). Retrospective analysis of clinical features in 134 coronavirus disease 2019 cases. *Epidemiol. Infect.* 148:e199. 10.1017/S0950268820002010 PMC7487751 32878654

[B66] Zhao J. Yang Y. Huang H.-P. Li D. Gu D.-F. Lu X.-F. (2020). Relationship between the ABO blood group and the COVID-19 susceptibility. *Clin. Infect. Dis.* 4:ciaa1150. 10.1093/cid/ciaa1150 32750119 PMC7454371

[B67] Zhao P. Praissman J. L. Grant O. C. Cai Y. Xiao T. Rosenbalm K. E. (2020). Virus-receptor interactions of glycosylated SARS-CoV-2 spike and human ACE2 receptor. *Cell Host. Microbe.* 28 586–601.e586. 32841605 10.1016/j.chom.2020.08.004 PMC7443692

[B68] Zietz M. Zucker J. Tatonetti M. P. (2020). Associations between blood type and COVID-19 infection, intubation, and death. *Nat. Commun.* 11:5761. 10.1038/s41467-020-19623-x PMC7666188 33188185

